# Prevalence of Major Depressive Disorder Among Adults in China: A Systematic Review and Meta-Analysis

**DOI:** 10.3389/fpsyt.2021.659470

**Published:** 2021-06-08

**Authors:** Yan-Jie Zhao, Yu Jin, Wen-Wang Rao, Qing-E Zhang, Ling Zhang, Todd Jackson, Zhao-Hui Su, Mi Xiang, Zhen Yuan, Yu-Tao Xiang

**Affiliations:** ^1^Unit of Psychiatry, Department of Public Health and Medicinal Administration, Institute of Translational Medicine, Faculty of Health Sciences, University of Macau, Macao, China; ^2^Centre for Cognitive and Brain Sciences, University of Macau, Macao, China; ^3^Institute of Advanced Studies in Humanities and Social Sciences, University of Macau, Macao, China; ^4^College of Education for the Future, Beijing Normal University, Zhuhai, China; ^5^The National Clinical Research Center for Mental Disorders & Beijing Key Laboratory of Mental Disorders, The Advanced Innovation Center for Human Brain Protection, Beijing Anding Hospital, Capital Medical University, Beijing, China; ^6^Department of Psychology, Faculty of Social Sciences, University of Macau, Macao, China; ^7^Center on Smart and Connected Health Technologies, Mays Cancer Center, School of Nursing, UT Health San Antonio, San Antonio, TX, United States; ^8^School of Public Health, Shanghai Jiao Tong University, Shanghai, China

**Keywords:** depression, Chinese, epidemiology, meta-analysis, prevalence

## Abstract

**Background:** Prevalence estimates of major depressive disorder (MDD) among adults in China have varied widely between studies. In this systematic review and meta-analysis, the overall prevalence of MDD in the Chinese population was estimated from published epidemiological studies and potential moderators that account for variability in estimates were assessed.

**Methods:** A systematic literature search was conducted in PubMed, EMBASE, Web of Science, PsycINFO, China National Knowledge Internet (CNKI), and WanFang databases to identify relevant studies. Data analyses were conducted using the Comprehensive Meta-Analysis Version 2.0.

**Results:** Forty studies comprising 1,024,087 subjects were included. The pooled point, 12-month, and lifetime prevalence rates of MDD in China were 1.1% (95% CI: 0.9–1.4%), 1.6% (95% CI: 1.0–2.5%), and 1.8% (95% CI: 1.5–2.2%), respectively. Subgroup and meta-regression analyses revealed gender, marital status, survey year, being published in English language, use of the Diagnostic and Statistical Manual of Mental Disorders (DSM) diagnostic systems and age as significant moderators of MDD prevalence.

**Conclusion:** The overall prevalence of MDD in the Chinese population appears to be lower than that of most countries, but the rates have been increasing over time and are elevated in particular demographic subgroups. Due to the negative consequences of MDD, effective preventive measures, early identification, and timely treatments are still important and should be offered to those in need.

## Introduction

Major depressive disorder (MDD) is a potentially severe psychiatric disorder associated with great personal suffering (1, 2) and enormous economic burdens on families and societies (3). During the past several decades, the epidemiology of MDD has been widely studied. For example, the Global Burden of Diseases, Injuries, and Risk Factors Study 2017 (GBD 2017) (4) found that 2% of the global population (approximately 163 million people) is affected by MDD.

MDD prevalence is also influenced by socio-cultural and economic factors (5). For example, in the World Mental Health (WMH) survey, the lifetime prevalence of MDD was 6.6% in Japan while the corresponding figure was 21.0% in France (5). Furthermore, the average lifetime prevalence of MDD (14.6%) in high income countries was elevated compared to the rate in low-middle income countries (11.1%) (5). Compared to men, women usually have a higher risk of MDD (5, 6). Older age (7) and unstable marital status (5, 8, 9) are also associated with higher likelihood of MDD. Because the epidemiology of MDD is strongly influenced by the particular socioeconomic context under examination (10–12), its epidemiology should be evaluated separately in different countries and regions or during different time periods relative to global estimates.

The prevalence of MDD has been studied in numerous China-based studies (13–17), but estimates have been highly inconsistent, varying from 0.2% (18) to 6.9% (19). Discrepancies between studies could be due to differences in survey periods, sampling, study sites, diagnostic criteria, and sample demographic characteristics. In order to reduce the negative outcomes of MDD, develop effective preventive measures, and allocate health resources for those at higher risk, it is necessary to understand the overall prevalence of MDD as well as changes in its rate over time and factors that contribute to variable rates. Evidence-based strategies such as systematic reviews and meta-analyses are efficient, timely approaches to addressing these issues.

One past meta-analysis (20) of 17 studies (total sample N = 176,435) found that the pooled 1-month, 12-month, and lifetime prevalence rates of MDD in mainland China were 1.6%, 2.3%, and 3.3%, respectively. Unfortunately, however, this review had important limitations including the failure to consider moderating factors (e.g., survey year, publication language, sample demographics), a lack of study quality assessment, and the exclusion of publication bias tests, all of which could affect the specificity or validity of findings. Therefore, we conducted this updated meta-analysis to examine the prevalence of MDD in the Chinese general population as well as potential methodological factors and demographic characteristics that contribute to variability in rates between studies and population subgroups.

## Methods

This meta-analysis was conducted according to the Preferred Reporting Items for Systematic Reviews and Meta-Analyses (PRISMA) (21), with the registration number of CRD42020184099 on PROSPERO.

### Literature Search and Selection

Three researchers (YJZ, YJ, WWR) independently and systematically conducted literature searches in PubMed, EMBASE, Web of Science, PsycINFO, China National Knowledge Internet (CNKI), and WanFang databases from their inception to September 26, 2019. The search strategy was developed based on the PICOS principle. Population (***P***): Chinese population; Intervention (***I***): not applicable; Comparators (***C***): not applicable; Outcome (***O***): lifetime, 1-year, and 1-month prevalence of MDD; Study design (***S***): epidemiological, cross-sectional surveys. The following search terms were used: “major depressi^*^,” “epidemiology,” “survey,” “prevalence,” “rate,” “percentage,” “China,” and “Chinese.” Manual search was also conducted by reviewing reference lists of retrieved articles for additional studies. The same three researchers independently screened titles and abstracts to identify potentially relevant articles, and then the full texts of potentially relevant articles were read for eligibility. If multiple papers were published based on the same dataset, only the one with the most complete data was included. Any disagreement was resolved by consensus.

Study inclusion criteria were: (1) reported prevalence of MDD, or relevant data that could generate prevalence of MDD. The diagnosis of MDD was made based on international or local diagnostic criteria, such as the *Diagnostic and Statistical Manual of Mental Disorders* (*DSM*), International Classification of Diseases (ICD), or Chinese Classification and Diagnostic Criteria of Mental Disorders (CCMD) system; (2) studies were conducted in mainland China; (3) reported prevalence timeframe, such as 1-year or 1-month prevalence. Exclusion criteria were: (1) case studies, reviews, systematic reviews, meta-analyses, commentaries; (2) studies conducted in special populations, such as adolescents, the elderly, women, or patients with chronic diseases.

### Data Extraction

Two researchers (YJZ and YJ) independently extracted data on participant and study characteristics, such as the first author, publication year, study location, survey period, sample size, sampling method, and prevalence of MDD. Any disagreement was resolved by consensus.

### Quality Assessment

Following other studies (22, 23), study quality was evaluated by Loney's 8-item scale (24) which includes the following domains: definition of the target population, sampling method, response rate, non-responder description, representativeness of samples, data collection method, diagnostic criteria, and precision of prevalence estimates. The total score ranged from 0 to 8. A total score of 7–8 was defined as “high quality,” while 4–6 scores were coded as “moderate quality” and 0–3 scores were rated as “low quality.” Two researchers (YJZ and YJ) independently evaluated study quality, and any disagreement was resolved by consensus or in consultation with the corresponding author (YTX).

### Data Analysis

Data analyses were performed with Comprehensive Meta-Analysis Version 2.0 (CMA V2.0, Biostat Inc., Englewood, New Jersey, USA). Percentage with MDD (%) represented individual effect sizes of included studies. *I*^2^ test was used to evaluate heterogeneity between studies. An *I*^2^> 50% indicates significant heterogeneity. Random-effects models were used in all analyses due to different basic demographic and clinical variables between studies. Subgroup analyses were conducted to explore moderating effects of categorical variables (i.e., publication language, diagnostic criteria, urban vs. rural living area, sex, education level, marital status) on prevalence rates. Sensitivity analyses were performed by removing each study one by one to explore potential sources of heterogeneity. Meta-regression analyses were performed to examine moderating effects of continuous variables (i.e., survey year, male percentage per sample, mean age, study quality rating score). Publication bias was examined via Egger's test and funnel plot inspection. Trim-and-fill analyses were performed to further assess the potential publication biases and generate adjusted overall rates after accounting for publication biases (25). Two-tailed *p*-values lower than 0.05 were considered as statistically significant.

## Results

### Study Characteristics

**Figure 1:**
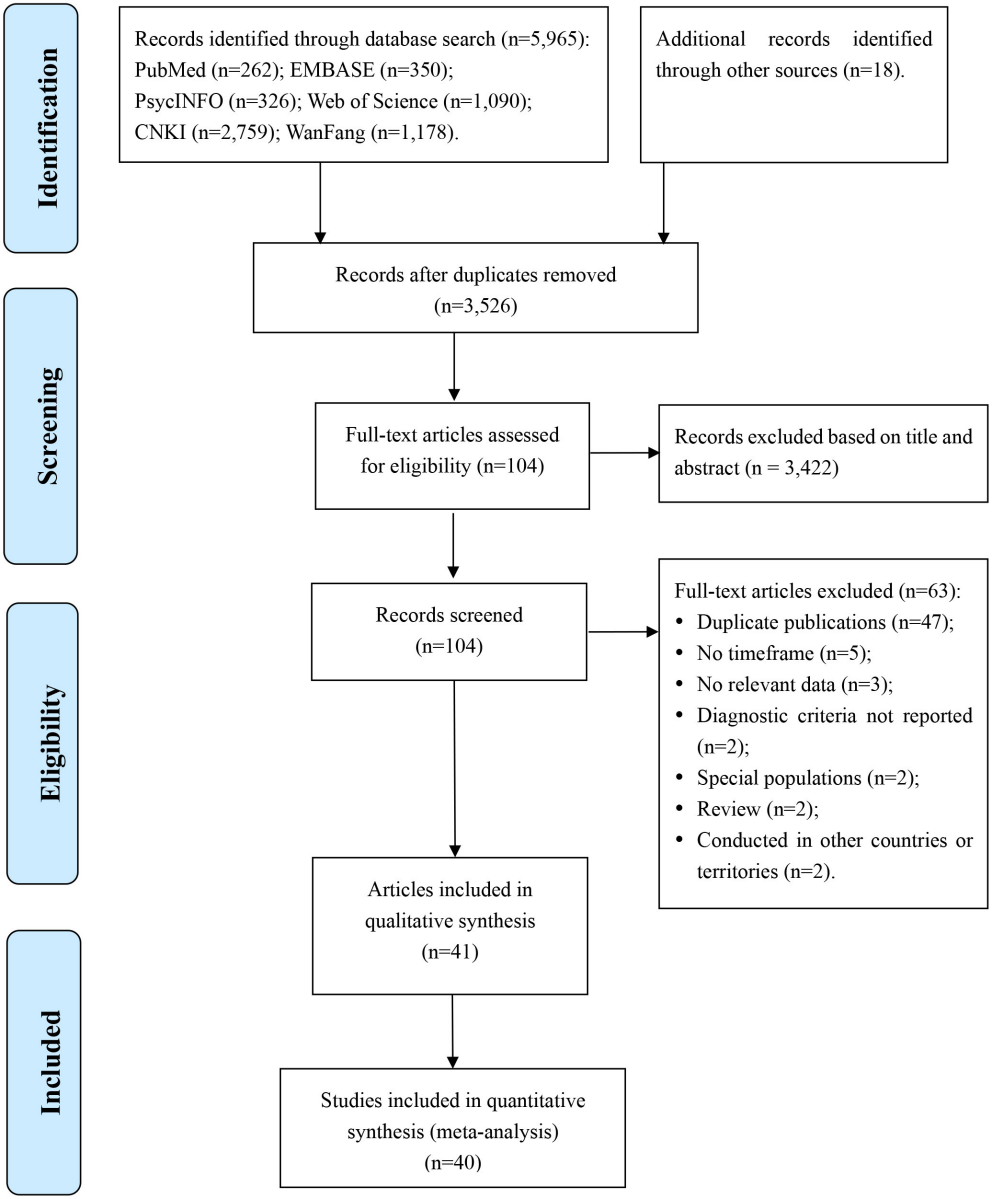
Flow diagram.

Of the 5,983 articles identified in the literature search, 40 studies from 41 articles involving 1,024,087 subjects were included in this meta-analysis. Two articles (26, 27) were based on one study, but one article reported subgroup details of lifetime prevalence (26) while the other reported subgroup details of 1-month prevalence (27). Therefore, both of them were included though analyses were conducted separately for lifetime, 1-year, and 1-month prevalence. Literature search, screening, and selection processes are displayed in Figure 1.

Study characteristics are presented in Table 1. Seven studies were published in English-language journals while the other 33 studies were published in Chinese-language journals. Twenty-nine studies used the *DSM*, 4 studies used the ICD, and 5 studies used the CCMD criteria while two studies used both ICD and CCMD criteria. Studies were conducted between 1982 and 2016 in 27 provinces or municipalities of China. The mean age of participants ranged from 32 to 55 years.

**Table 1:** Characteristics of studies included in this meta-analysis.

**First Author**	Publication Language	Survey Time	Study Site	Living Area	Diagnostic Criteria	Diagnostic Tool	**Age**				**Total Sample**	**Female (%)**	**Lifetime Events**	**12-Month Events**	**Point Events**	**Quality Evaluation**	**Response Rate (%)**	**Sampling Method**	**References**
							**Min**	**Max**	**Mean**	**SD**									
Zhao et al. (28)	Chinese	1982.7	12 districts	Both	ICD-9	NR	15	70+	NR	NR	38,136	49.73	10	NR	NR	5	NR	CR	(28)
Wang et al. (18)	Chinese	1993.4	7 provinces	Both	ICD-9 and CCMD-2	NR	15	60+	NR	NR	19,223	50.04	4	NR	2	5	NR	CR	(18)
Xiao et al. (29)	Chinese	NR	Shanghai, Yangpu	Urban	CCMD-II-R	NR	16	60	NR	NR	38,80	49.36	NR	NR	26	5	96.6	R	(29)
Hu et al. (30)	Chinese	2002.3–4	Jiangxi	Both	ICD-10	CIDI	15	65+	NR	NR	15,939	49.76	81	NR	56	7	87	SCR	(30)
Guo et al. (31)	Chinese	2004.4–11	Shanxi, Xian	Fringe area	CCMD-3	NR	16	59	NR	NR	1,953	50.49	NR	NR	23	5	96.9	C	(31)
Ma et al. (19)	Chinese	2003.4	Beijing	Both	ICD-10	CIDI-1.0	15	65+	NR	NR	5,926	53.85	407	NR	196	8	82.3	MSR	(19)
Cui et al. (32)	Chinese	2004.10–2005.3	Hebei	Both	*DSM-IV*	SCID-I/P	18	95	44	15	20,716	50.07	608	NR	399	7	86.3	MSR	(32)
Zhang et al. (33)	Chinese	2004.11–2005.4	Liaoning	Both	*DSM-III-R*	CIDI-1.0	18	65	41.39	NR	13,358	50.52	345	245	NR	7	86.1	MSR	(33)
Dong et al. (34)	Chinese	2006.9–2007.2	Shandong, Weihai	Both	CCMD-3	NR	15	60+	NR	NR	50,174	49.50	1,881	NR	1,393	5	99.9	SCR	(34)
Phillips et al. (35)	English	2001.9–2005.12	4 provinces	Both	*DSM-IV*	SCID	18	NR	NR	NR	63,004	51.23	NR	NR	1,034	8	94.7	MSR	(35)
Lee et al. (36)	English	2001.11–2002.2	Beijing; Shanghai	Urban	*DSM-IV*	CIDI	18	80	NR	NR	5,201	51.30	181	89	NR	6	74.7	MC	(36)
Zhao et al. (37)	Chinese	2006.8	Guangdong, Guangzhou	Both	*DSM-IV*	SCID-I/P	15	99	47.3	17.2	7,418	56.51	342	NR	62	7	92.7	SCR	(37)
Gui et al. (38)	Chinese	2007.1–5	Hunan, Liuyang	Rural	*DSM-IV*	SCID-I/P	15.00	97.25	49.73	16.30	7,347	54.29	234	NR	140	7	69.3	MSR	(38)
Li et al. (39)	Chinese	2005.2	Jiangsu, Xuzhou	Urban	CCMD-3	NR	15	60	NR	NR	4,299	47.92	37	NR	29	5	96.8	R	(39)
Duan et al. (40)	Chinese	2005.9–12	Guangdong, Shenzhen	NR	*DSM-IV*	CIDI-3.1	18	NR	32.49	11.16	7,134	49.33	429	215	90	5	79.9	MSR	(40)
Peng et al. (41)	Chinese	2009.6	Hunan, Hengyang	Both	CCMD-2-R	NR	15	50+	NR	NR	4,298	42.81	3	NR	NR	3	NR	NR	(41)
Yao et al. (42)	Chinese	2005.11–2006.1	Yunnan, Kunming	Both	*DSM-IV*	CIDI-2.1	15	NR	39.05	15.17	5,033	52.00	79	57	42	6	99.5	MSR	(42)
Fang et al. (43)	Chinese	2009.2–6	Fujian	Both	*DSM-IV*	SCID-I/P	15	NR	41.3	16.6	9,986	49.14	NR	NR	186	8	99.9	MSCR	(43)
Wei et al. (44)	Chinese	2007.7–12	Guangxi	Both	ICD-10	CIDI-3.0	15	107	42.04	16.58	18,219	49.53	97	NR	62	8	85.6	MSCR	(44)
Liu et al. (45)	Chinese	2009.12–2010.1	Yunnan, Jinuo	Rural	*DSM-IV-TR*	SCID-I/P	15	88	40	16	1,977	47.04	NR	NR	47	8	94.1	R	(45)
Chen et al. (46)	English	2010	Beijing	Both	*DSM-IV*	CIDI-3.0	16/18	97	NR	NR	2,469	60.83	NR	81	NR	5	72.9	M	(46)
Liu et al. (47)	Chinese	2009.9–2010.3	Sichuan, Yibin	Both	*DSM-IV*	SCID	18	98	49	15	11,227	53.17	67	NR	39	7	93.6	MSCR	(47)
Wang et al. (27)	Chinese	2010.5–11	Fujian, Xiamen	Both	*DSM-IV*	SCID-I/P	18	98	43	16	10,764	53.95	145	NR	63	8	89.2	MSCR	(27)
Liao et al. (26)	Chinese	2010.5–11	Fujian, Xiamen	Both	*DSM-IV*	SCID-I/P	NR	NR	NR	NR	10,764	NR	145	NR	63	6	89.2	MSCR	(26)
Yu et al. (16)	Chinese	2004–2008	10 provinces	Both	*DSM-V*	CIDI-A	30	79	51.5	10.7	512,891	59.01	NR	3281	NR	5	NR	NR	(16)
Liu et al. (13)	English	2010.11–12	Beijing	Both	*DSM-IV-TR*	SCID-I/P	18	75+	NR	NR	16,032	50.62	571	NR	176	8	80.7	MSCR	(13)
Zhang et al. (48)	Chinese	2014.10–12	Hubei, Jingzhou	Rural	*DSM-III-R and DSM-IV*	CIDI	15	75	47.8	12.7	249	36.55	9	2	1	5	NR	CR	(48)
Shi et al. (49)	Chinese	2010.5–8	Shanxi, Xian	Both	*DSM-IV*	CIDI-3.0	16	75	46.7	13.2	2,447	61.18	95	43	12	6	68.4	MSR	(49)
Ren et al. (50)	Chinese	2014.1–2015.1	Beijing, Huilongguan	Fringe area	ICD-10 and CCMD-3	NR	6	80	NR	NR	34,625	45.00	1,727	NR	NR	4	NR	Census	(50)
Ou et al. (51)	Chinese	2015	Guangdong, Huizhou	Both	*DSM-IV*	NR	18	NR	NR	NR	2,400	50.46	NR	NR	113	5	100	MSCR	(51)
Wang Z. et al. (52)	Chinese	2014.10–2015.6	Liaoning	Both	*DSM-IV*	SCID-I/P	18	80+	52.64	15.93	19,733	55.79	NR	NR	471	7	82.2	MSCR	(52)
Chen et al. (53)	Chinese	2011.7–12	Tianjin	NR	*DSM-IV*	SCID-I/P	18	NR	NR	NR	11,748	NR	439	NR	NR	6	75.6	MCR	(53)
Li et al. (54)	Chinese	2015.10–2016.5	Shandong, Liaocheng	Both	*DSM-IV*	SCID-I/P	18	88	42.00	2.68	1,799	50.92	41	NR	40	7	99.9	MSCR	(54)
Zhao et al. (55)	Chinese	2015.10–2016.5	Shandong, Linyi	Both	*DSM-IV*	SCID-I/P	18	98	54.51	16.17	3,670	58.77	NR	NR	83	8	94.1	SR	(55)
Ge et al. (56)	Chinese	2015.11–2016.4	Shandong, Weifang	Both	*DSM-IV*	SCID-I/P	18	93	55.36	14.12	4,797	56.26	NR	NR	105	7	99.6	MSCR	(56)
Xu L. et al. (57)	Chinese	2016.10	Yunnan, Mosuo	Rural	*DSM-V*	MINI and SCID-I/P	15	88	46.5	12.2	1,121	66.28	NR	NR	20	7	99.8	MSR	(57)
Huang et al. (58)	English	2013.7–2015.3	31 provinces	Both	*DSM-IV*	CIDI 3.0 and SCID	18	65+	NR	NR	32,552	54.58	1,093	655	NR	8	84.3	MC	(58)
Yue et al. (59)	Chinese	2010.9–2011.11	Hainan	Both	*DSM-IV*	SCID-I/P	15	99	42	16	12,117	46.83	97	NR	33	7	100.9	MSCR	(59)
Zhang et al. (17)	English	2016.4–8	Hebei	Both	*DSM-IV*	SCID-I/P	18	98	48.87	16.14	20,884	51.25	499	NR	289	8	88.2	MSCR	(17)
Jacob et al. (60)	English	2007–2010	national	Both	*DSM-IV*	CIDI	18	114	45.6	12.8	14,813	NR	NR	123	NR	6	93	MC	(60)
Cui et al. (61)	Chinese	2010–2011	Neimenggu, Chifeng	Both	*DSM-IV*	CIDI-3.0-CAPI	18	65+	NR	NR	4,528	53.95	234	95	NR	6	71.0	SR	(61)

*CCMD, Chinese Classification of Mental Disorder; CIDI, Composite International Diagnostic Interview; DSM, Diagnostic and Statistical Manual of Mental Disorders; SCID, Structured Clinical Interview for DSM; ICD, International Classification of Disease; MINI, Mini-International Neuropsychiatric Interview; NR, not reported; M, multistage; SD, standard deviation; S, stratified; C, cluster; R, random*.

*Point events included 1-month, 2-week, and timepoint events*.

### Pooled Prevalence of MDD in China

**Figure 2:**
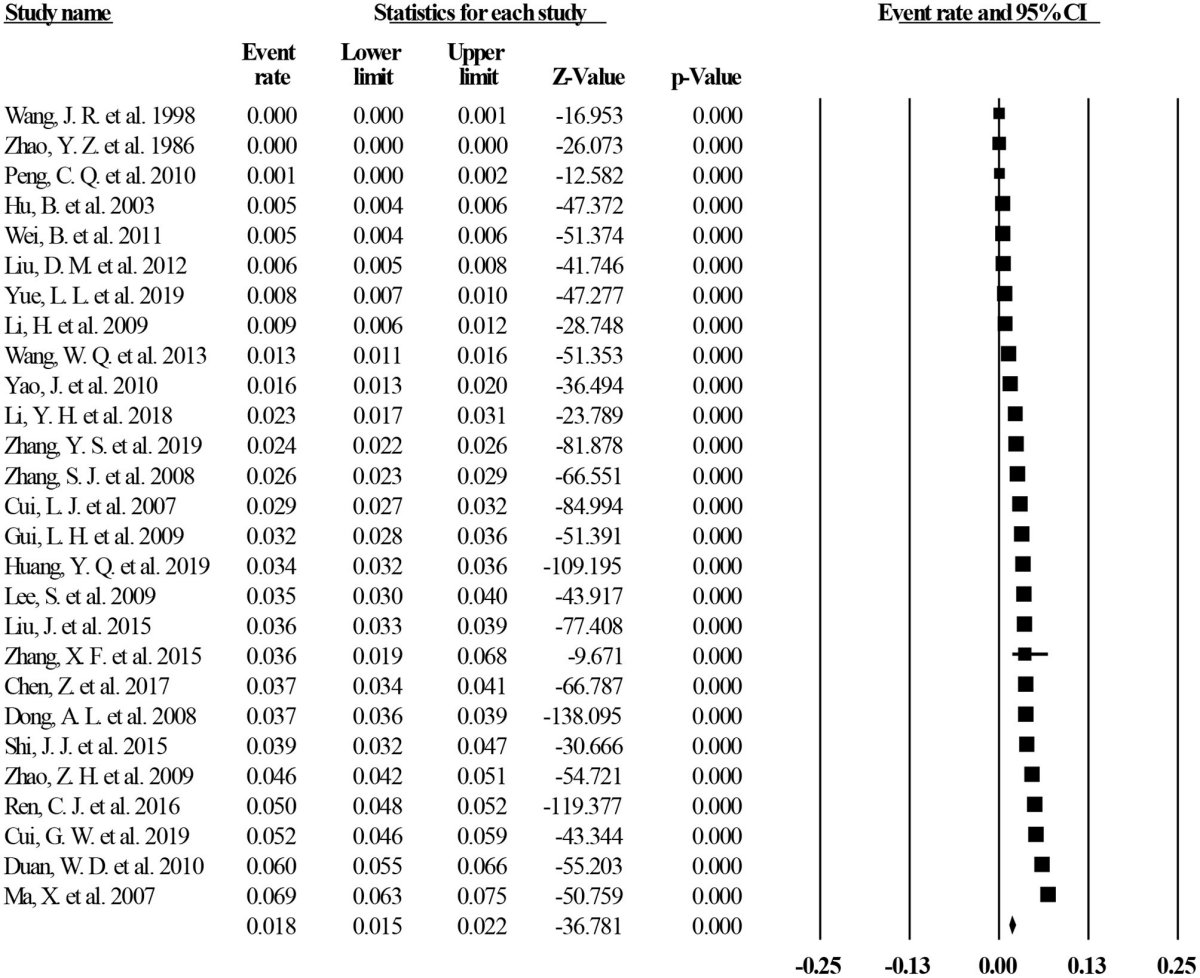
Lifetime prevalence of MDD in China.

**Figure 3:**
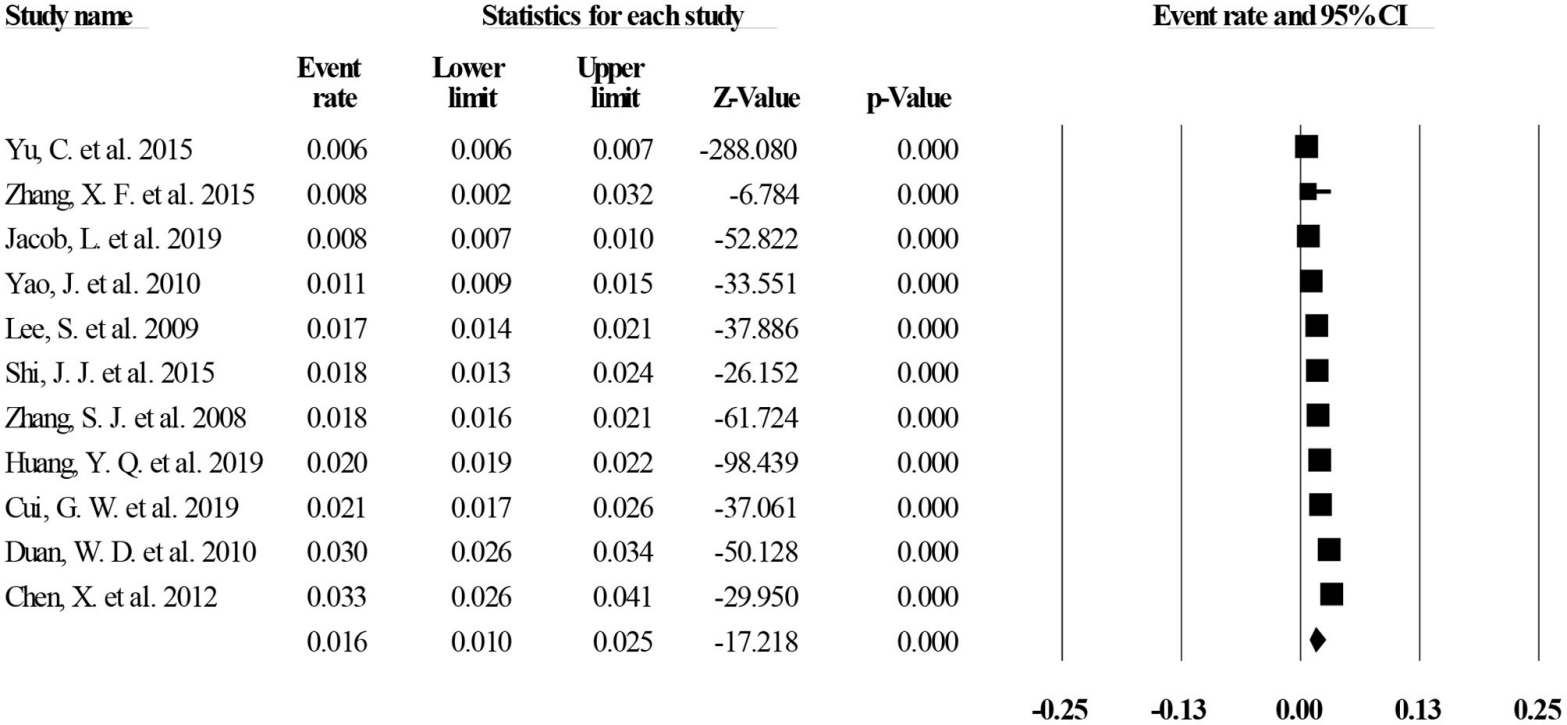
Twelve-month prevalence of MDD in China.

**Figure 4:**
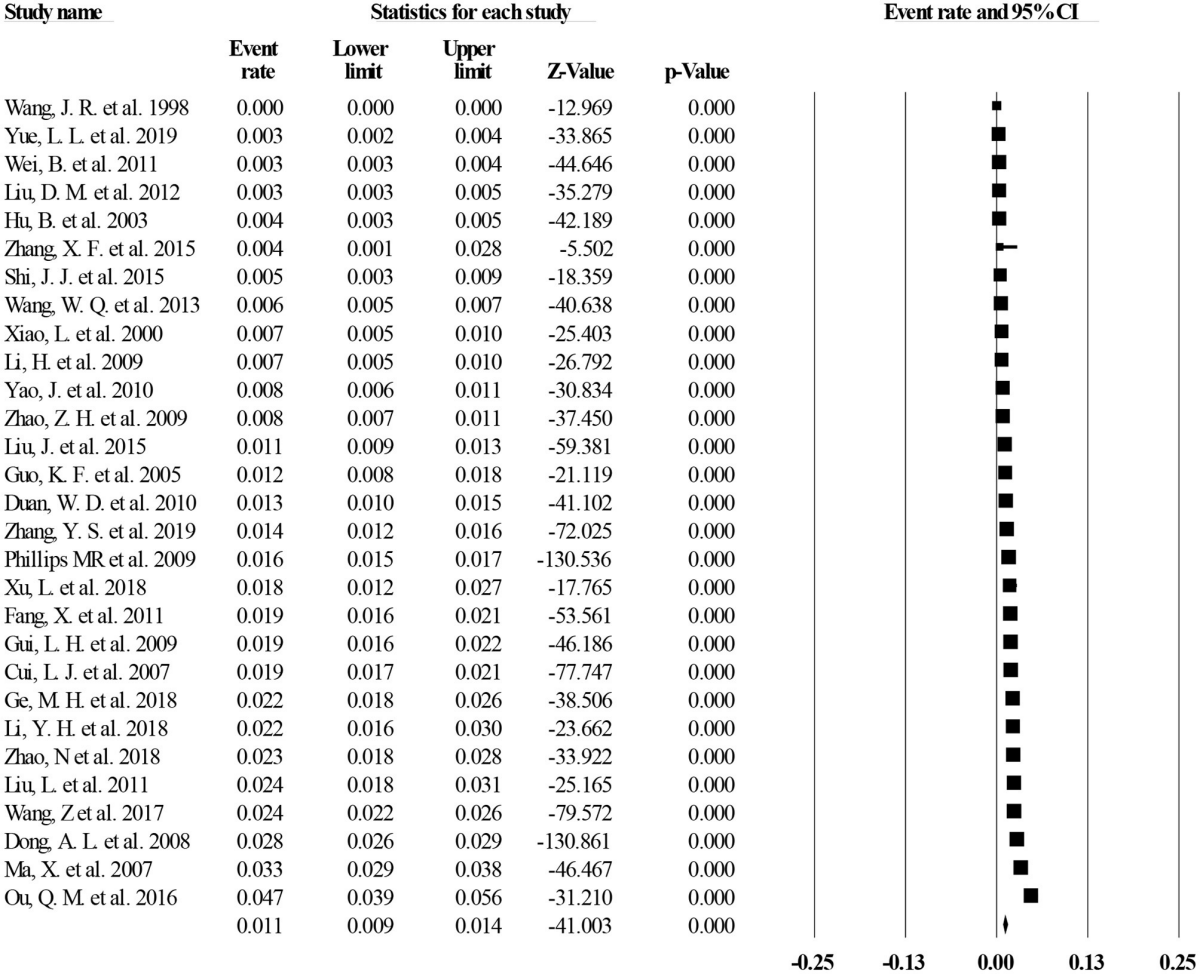
Point prevalence of MDD in China.

Of the 40 studies, 27 reported lifetime prevalence of MDD; based on this subset, the pooled lifetime prevalence of MDD was 1.8% (95% CI: 1.5–2.2%, *I*^2^ = 98.97%, *p* < 0.001; Figure 2). Based on 11 studies that reported the 12-month prevalence of MDD, the pooled 12-month prevalence rate was 1.6% (95% CI: 1.0–2.5%, *I*^2^= 99.34%, *p* < 0.001; Figure 3). Finally, based on 29 studies that reported point prevalence of MDD, the pooled point prevalence rate was 1.1% (95% CI: 0.9–1.4%, *I*^2^ = 98.01%, *p* < 0.001; Figure 4). In sensitivity analyses, no significant changes were found after included studies were removed individually.

### Subgroup Analyses

Subgroup analyses revealed that a higher lifetime prevalence of MDD was associated with studies published in English language and studies using *DSM* diagnostic systems (both *p* < 0.001). For sex, women had a higher point prevalence of MDD (*p* = 0.001) though sex differences were not significant for 1-year or lifetime prevalence. Marital status was significantly associated with both lifetime and point prevalence of MDD (both *p* < 0.001). Divorced and remarried participants had the highest MDD prevalence (10.0% for lifetime and 5.4% for point prevalence), followed by widowed participants (5.7% for lifetime and 2.5% for point prevalence), married participants (2.3% for lifetime and 1.1% for point prevalence), and never-married cohorts (1.4% for lifetime and 0.7% for point prevalence) (Tables 2, 3). There were no significant moderating effects for rural vs. urban residence or education level on the primary results (Tables 2–4).

**Table 2:** Subgroup analyses of lifetime prevalence of MDD in China.

**Subgroups**	**Categories**	**Number of studies**	**Events**	**Sample size**	**Prevalence (%)**	**95%** ***CI***		***I^**2**^* (%)**	***p* (within subgroup)**	***Q* (*p* across subgroups)**
Publication language	Chinese	23	7,411	306,724	1.6	1.2	2.0	99.10	<0.001	*Q* = 18.88 *p* <0.001
	English	4	2,344	74,669	3.2	2.6	3.8	94.50	<0.001	
Diagnostic criteria	*DSM*	18	5,508	190,554	2.7	2.2	3.3	98.14	<0.001	*Q* = 6.16 *p* = 0.046
	ICD	4	595	78,220	0.5	0.1	3.2	99.72	<0.001	
	CCMD	3	1,921	58,771	0.7	0.2	3.0	98.45	<0.001	
Living area	Rural	11	30,82	99,352	2.6	1.9	3.6	98.61	<0.001	*Q* = 0.87 *p* = 0.35
	Urban	11	1,834	76,174	2.0	1.4	3.0	98.47	<0.001	
Sex	Male	16	2,118	119,570	1.6	1.1	2.1	97.66	<0.001	*Q* = 3.23 *p* = 0.07
	Female	16	3,581	122,039	2.3	1.7	3.0	98.50	<0.001	
Education level	Primary school or below	6	676	26,555	2.6	1.2	5.8	98.99	<0.001	*Q* = 0.71 *p* = 0.87
	Junior high school	6	583	30,160	1.8	1.0	3.6	98.38	<0.001	
	Senior high school	6	360	15,653	1.7	0.8	3.6	97.77	<0.001	
	College or above	6	349	8,671	1.8	0.6	5.2	98.08	<0.001	
Marital status	Married	5	1,535	60,860	2.3	1.3	4.1	99.17	<0.001	*Q* = 39.15 *p* <0.001
	Never married	5	114	8,905	1.4	0.8	2.6	90.70	<0.001	
	Divorced or remarried	5	102	1,003	10.0	7.1	14.0	68.92	0.012	
	Widowed	5	200	3,241	5.7	3.5	9.1	90.99	<0.001	

*CI, confidence interval*.

**Table 3:** Subgroup analyses of point prevalence of MDD in China.

**Subgroups**	**Categories**	**Number of studies**	**Events**	**Sample size**	**Prevalence (%)**	**95%** ***CI***		***I^**2**^* (%)**	***p* (within subgroup)**	***Q* (*p* across subgroups)**
Publication language	Chinese	26	3,733	249,548	1.1	0.8	1.4	98.05	<0.001	*Q* = 1.82 *p* = 0.18
	English	3	1,499	99,920	1.4	1.1	1.7	92.77	<0.001	
Diagnostic criteria	*DSM*	21	3,445	229,855	1.3	1.1	1.6	96.77	<0.001	*Q* = 0.58 *p* = 0.75
	ICD	3	314	40,084	0.7	0.1	3.9	99.47	<0.001	
	CCMD	4	1,471	60,306	1.1	0.5	2.7	97.58	<0.001	
Living area	Rural	15	1,656	84,445	1.7	1.0	2.9	99.02	<0.001	*Q* = 3.70 *p* = 0.054
	Urban	13	700	76,981	0.9	0.6	1.4	96.90	<0.001	
Sex	Male	19	814	81,399	0.9	0.7	1.1	91.57	<0.001	*Q* = 10.64 *p* = 0.001
	Female	19	1,624	88,276	1.6	1.2	2.0	95.20	<0.001	
Education level	Primary school or below	4	300	15,636	1.6	0.6	4.2	98.06	<0.001	*Q* = 2.25 *p* = 0.52
	Junior high school	4	173	18,674	1.0	0.5	2.1	95.39	<0.001	
	Senior high school	4	111	11,090	1.0	0.5	2.0	92.71	<0.001	
	College or above	4	52	6,704	0.6	0.2	1.6	88.10	<0.001	
Marital status	Married	3	416	36,372	1.1	0.4	2.9	98.91	<0.001	*Q* = 19.58 *p* <0.001
	Never married	3	33	5,856	0.7	0.2	2.0	90.32	<0.001	
	Divorced or remarried	3	26	496	5.4	3.7	7.8	0	0.416	
	Widowed	3	65	2,221	2.5	1.1	5.7	88.99	<0.001	

*CI, confidence interval*.

**Table 4:** Subgroup analyses of 12-month prevalence of MDD in China.

**Subgroups**	**Categories**	**Number of studies**	**Events**	**Sample size**	**Prevalence (%)**	**95%** ***CI***		***I^**2**^* (%)**	***p* (within subgroup)**	***Q* (*p* across subgroups)**
Publication language	Chinese	7	3,938	545,640	1.5	0.8	2.8	99.28	<0.001	*Q* = 0.17 *p* = 0.68
	English	4	948	55,035	1.8	1.1	2.8	97.34	<0.001	
Living area	Rural	5	624	25,326	2.5	2.0	3.1	77.23	<0.001	*Q* = 0.38 *p* = 0.54
	Urban	5	513	26,807	2.2	1.6	3.1	91.55	<0.001	
Sex	Male	9	1,500	244,219	1.5	0.9	2.7	98.79	<0.001	*Q* = 0.40 *p* = 0.53
	Female	9	3,258	341,394	1.9	1.2	3.3	99.16	<0.001	
Education level	Primary school or below	3	45	1,361	3.4	2.2	5.2	47.47	0.149	*Q* = 0.37 *p* = 0.95
	Junior high school	3	99	3,043	3.7	2.1	6.2	86.23	0.001	
	Senior high school	3	130	3,536	4.0	2.7	6.0	77.76	0.011	
	College or above	3	129	3,242	3.8	2.7	5.2	54.34	0.112	

*Diagnostic criteria were not analyzed in the subgroup analysis because DSM criteria were used in all 11 studies included. CI, confidence interval*.

### Meta-Regression Analyses

In meta-regression analyses both lifetime and point prevalence of MDD had significant positive associations with more recent survey dates (*r* = 0.125 for lifetime, *p* <0.001; *r* = 0.09 for point prevalence, *p* <0.001), while lifetime prevalence of MDD was negatively associated with percentage of males per sample (*r* = −7.16, *p* = 0.01). Studies with higher quality reported higher lifetime prevalence of MDD (*r* = 0.23, *p* = 0.009). Finally, mean age of the participants was negatively related with the 12-month prevalence of MDD (*r* = −0.07, *p* <0.001; Supplementary Table 2). Moderating effects of other continuous demographics on MDD prevalence were not significant.

### Quality Assessment and Publication Bias

The mean quality assessment score was 6.4 and ranged from 3 to 8. Of the 41 articles, 21 (51.2%) were rated high quality, 19 (46.3%) were rated moderate quality, and 1 (2.4%) was rated low quality (Supplementary Table 1). Egger's tests found that publication bias existed in studies on lifetime, 12-month, and point prevalence of MDD (all *p* <0.05), as shown in Table 5. The Duval and Tweedie trim-and-fill analyses suggested that 10, 6, and 11 studies with higher or lower than average prevalence estimates might be missing from effect size distributions and would need to be imputed to achieve approximately symmetrical funnel plots of lifetime, 12-month, and point prevalence of MDD, respectively. Using trim-and-fill analyses, imputed estimates for lifetime, 12-month, and point prevalence of MDD would change to 3.8% (95% CI: 3.0–4.8%), 0.7% (95% CI: 0.5–1.2%), and 2.0% (95% CI: 1.6–2.5%), respectively. Funnel plots and imputed funnel plots were shown in Supplementary Figures 1–6.

**Table 5:** Pooled prevalence of MDD in China.

**Timeframe**	**Number of studies**	**Events**	**Sample size**	**Prevalence (%)**	**95%** ***CI***		***I^**2**^* (%)**	***p***	**Publication bias (Egger's test)**
Lifetime prevalence	27	9,755	381,393	1.8	1.5	2.2	98.97	<0.001	*t* = 4.17, *p* <0.001
12-month prevalence	11	4,886	600,675	1.6	1.0	2.5	99.34	<0.001	*t* = 2.53, *p* = 0.032
Point prevalence	29	5,232	349,468	1.1	0.9	1.4	98.01	<0.001	*t* = 3.69, *p* = 0.001

*I^2^statistic was used to assess the heterogeneity of the studies*.

*Point prevalence included 1-month, 2-week, and timepoint prevalence. CI, confidence interval*.

## Discussion

To the best of our knowledge, this systematic review and meta-analysis included the largest number of studies and largest overall sample size estimating MDD prevalence in the Chinese population to date. The point prevalence in this meta-analysis was 1.1% (95% CI: 0.9–1.4%), which is significantly lower than the corresponding findings of previous meta-analyses in other countries; for instance, the point prevalence was 4.1% (95% CI: 3.1–5.5%) in a meta-analysis of MDD in Iran (62). Similarly, the overall 12-month prevalence (1.6%; 95% CI: 1.0–2.5%) and lifetime prevalence (1.8%; 95% CI: 1.5–2.2%) estimates of MDD in this meta-analysis were noticeably lower than rates from previous studies; for instance, in the World Mental Health Survey (WMH) the mean 12-month prevalence of MDD was 5.9% and ranged from 3.8% to 10.4% while the mean lifetime prevalence of MDD was 11.1%, and ranged from 6.5% to 18.4% in low-middle income countries (5).

Several factors might be responsible for the lower prevalence of MDD in this meta-analysis. In general, patients with psychiatric disorders including depression in China are ashamed of reporting their symptoms or feelings to others due to the stigma of mental illness and the fear of discrimination (63, 64). In addition, several authors have argued that Chinese patients with depression are more likely to somatize their distressing symptoms, which could lead to underestimated prevalence of psychiatric disorders (65–68).

Notably, however, MDD estimates from this meta-analysis were also lower than those generated from a previous meta-analysis on MDD in China (point prevalence: 1.6%, 95% CI: 1.2–1.9%; 12-month prevalence: 2.3%, 95% CI: 1.8–3.4%; lifetime prevalence: 3.3%, 95% CI: 2.4–4.1%) (20). Several epidemiological studies reporting very low MDD prevalence (18, 28, 41) were overlooked in Gu et al.'s meta-analysis and may have biased their findings, though, unfortunately, effects of publication biases were not evaluated in that review. In addition, compared to Gu et al.'s meta-analysis, 23 additional studies were included in this meta-analysis, increasing the statistical power of the findings. Finally, publication bias analyses from this review suggested that the number of studies with higher point and lifetime prevalence estimates may be underrepresented in the updated literature on MDD rates in Chinese samples. Trim and fill analyses suggested that point and lifetime prevalence estimates increase substantially when missing studies are considered in analyses and converge more closely with estimates from other countries and preliminary data from China.

Similar to previous studies (6, 69), both subgroup and meta-regression analyses revealed that women were more likely to suffer from MDD, a finding that may be attributed, in part, to hormonal influences in women (70, 71) and/or culturally sanctioned norms that encourage women to express depressive feelings and to seek professional help for distress (72–75). Associations between prevalence of MDD and age have been mixed in past work. Some researchers have proposed a U-shaped relationship between age and MDD prevalence, wherein adolescents and the elderly are high-risk populations for depression (76, 77). In other studies, such as the WHO World Mental Health Survey, elderly populations had a lower 12-month prevalence than did younger populations in developed countries (7). In this meta-analysis, older mean age of the participants was associated with lower 12-month prevalence of MDD. Due to their sometimes poor health status and more frequent negative life events such as retirement and increased risk of physical diseases, the elderly might be expected to have a higher risk of depression. However, the elderly are usually psychologically protected by social support systems and social welfare policies and often live with their families, all of which may reduce the risk of depression (36, 78, 79).

We found that rural residents had a higher lifetime, 12-month, and point prevalence of MDD than those living in urban areas, although differences were not statistically significant. This is consistent with results from the previous meta-analysis of MDD prevalence in China (point prevalence: 2.0% [95% CI: 1.2–2.9%] in rural areas, and 1.7% [95% CI: 0.8–2.7%] in urban areas) (20). Epidemiological studies have consistently found that urbanization level is negatively associated with the risk of depression (80, 81). However, some studies in other countries such as in the United States (82), Canada (83), the Netherlands (84), and Malaysia (85) found that urban residents had a higher risk developing depression compared to their rural counterparts. These discrepancies might be attributed to different cultural environments and sample differences in socioeconomic status (86–88). For instance, unlike in developed countries, primary care services in rural areas of China are greatly underdeveloped in rural areas, which could increase the likelihood of depression in rural regions.

For marital status, divorced or remarried subgroups had the highest prevalence of MDD compared with other marital status subgroups in this meta-analysis, in line with previous studies (89–91). We also found that the never married had the lowest point and lifetime prevalence of MDD of any marital status subgroups. However, this latter finding contrasts with evidence from data from the United States, Canada, and Japan, wherein the prevalence of depression among the never married has been higher than the rate among those who are married (92–95), potentially because the depressed are less likely to get married (96, 97). Variability between countries in associations between marital status and depression prevalence underscore culture as a potent influence on patterns of variability (5). We speculate that marriage-related conflicts and family-supporting pressure may increase risk for depression in married rather than never-married subgroups in Chinese samples.

Meta-regression analyses revealed that survey year was positively associated with lifetime and point prevalence of MDD; this is a novel finding that was not reported in the previous meta-analysis from China (20). Traditionally, Chinese people with depression tend to somatize their depressive symptoms due to perceived stigma and social discrimination related to mental illness (66, 98–100). With recent social developments and public education to increase awareness of psychiatric disorders, particularly depression, in China, prejudice to mental illness has been decreasing; thus, people with MDD may be more willing to express their depressive feelings and to seek help, which could result in increased prevalence of MDD (101, 102).

Higher study quality was also associated with higher lifetime prevalence of MDD. Stringent methodologies that include random sampling, large sample sizes, and, especially, strict training of interviewers in the appropriate conduct of diagnostic assessments are more typically used in high quality studies (103) and potentially increase the capacity to identify depressed patients, hence increasing reported MDD prevalence rates. Similarly, in this meta-analysis studies published in English-language journals reported a higher lifetime prevalence of MDD compared to those published in Chinese journals. Given that studies published in English language journals also had higher study quality assessment scores (mean score of 7) than those in Chinese journals (mean score of 6.2), it is possible that increased methodological rigor in studies from the former group facilitated the capacity to identify participants with MDD.

Previous meta-analyses of MDD prevalence have found that the ICD is the most sensitive diagnostic tool in identifying MDD in Chinese older adults or adolescents (104, 105), though the prevalence of MDD based on *DSM*, ICD, and CCMD criteria is often similar in the general population (106–108). In this meta-analysis, studies using the *DSM* diagnostic criteria reported significantly higher lifetime prevalence of MDD than those using the ICD or CCMD criteria. The larger proportion of included studies using the *DSM* (*n* = 18) vs. the ICD (*n* = 4) or CCMD (*n* = 3) suggests rates generated from the latter two systems may be less reliable because they are based on disproportionately fewer studies. In addition, most studies using the ICD or CCMD were conducted before 2010, while most studies using the *DSM* were conducted after 2010. As such, the above-mentioned positive relationship between prevalence of MDD and survey year may have also contributed to the moderating effect of diagnostic criteria on lifetime prevalence of MDD.

Strengths of this meta-analysis included the large number of studies and very large overall sample size as well as the inclusion of additional analyses designed to evaluate potential moderating influences on MDD rates, study quality assessment, and publication biases. Several methodological limitations should be noted. First, there was significant heterogeneity in results between studies. Such variability is often unavoidable in meta-analyses of epidemiological studies based on different participant characteristics, sampling methods, and assessment instruments found between studies (109, 110). Fortunately, moderator analyses were useful in identifying sociodemographic factors that contributed to heterogeneity in addition to at-risk subgroups (e.g., divorced, remarried, widowed, younger) that can benefit potentially from targeted outreach efforts. Second, certain factors associated with epidemiology of MDD, such as occupational status, family history of MDD, and social support, were not analyzed due to insufficient data. Third, all the included studies were based on cross-sectional designs so within sample changes in MDD rates over time and causal effects of other factors on MDD rates could not be determined. Fourth, only published data were synthesized. Unpublished data that were not included in the target databases were inaccessible and therefore were not included.

In conclusion, the prevalence of MDD in the general populations in China appeared to be lower than rates from meta-analyses from other countries though there was a trend toward increasing rates over time. Moderator analyses suggested that certain demographic subgroups including women and divorced, remarried, or widowed persons have comparatively higher levels of MDD and could benefit from targeted interventions. However, despite the low prevalence of MDD in China, considering the negative health impact and adverse consequences of MDD on quality of life and other outcomes, effective preventive measures, early identification, and timely treatments, particularly within at risk demographic groups, remain important and should be offered to those in need.

## Data Availability Statement

The original contributions presented in the study are included in the article/Supplementary Material, further inquiries can be directed to the corresponding author/s.

## Author Contributions

Y-TX: study design. Y-JZ, YJ, W-WR, Q-EZ, and LZ: collection, analysis, and interpretation of data. Y-JZ, YJ, and Y-TX: drafting of the manuscript. TJ and Z-HS: critical revision of the manuscript. All authors approved the final version for publication.

## Conflict of Interest

The authors declare that the research was conducted in the absence of any commercial or financial relationships that could be construed as a potential conflict of interest.
