# Supplementary material for: Prevalence of Major Depressive Disorder Among Adults in China: A Systematic Review and Meta-Analysis
Source: Front Psychiatry. 2021 Jun 8;12:659470. doi: 10.3389/fpsyt.2021.659470 (PMC8219051; doi:10.3389/fpsyt.2021.659470)
Supplement: Supplementary file 1 [file Data_Sheet_1.docx]

**Supplementary Table 1. Quality assessment of included studies.**

|  | **1. Target population is clearly defined** | **2. Probability sampling OR entire population surveyed** | **3. Is the response rate ≥80%?** | **4. Are non-responders clearly described?** | **5. Is the sample representative of the target population?** | **6. Were data collection methods standardized?** | **7. Were validated criteria used to diagnose MDD?** | **8. Are the prevalence estimates given with confidence intervals and detailed by subgroups**  **(if applicable) ?** | **Total score** |
| --- | --- | --- | --- | --- | --- | --- | --- | --- | --- |
|  |  |  |  |  |  |  |  |  |  |
| Yu, C. et al. 2015 | 1 | 0 | 0 | 0 | 1 | 1 | 1 | 1 | 5 |
| Huang, Y. Q. et al. 2019 | 1 | 1 | 1 | 1 | 1 | 1 | 1 | 1 | 8 |
| Phillips MR et al. 2009 | 1 | 1 | 1 | 1 | 1 | 1 | 1 | 1 | 8 |
| Wang, J. R. et al. 1998 | 1 | 1 | 0 | 0 | 1 | 1 | 1 | 0 | 5 |
| Chen, X. et al. 2012 | 1 | 1 | 0 | 0 | 1 | 1 | 1 | 0 | 5 |
| Liu, J. et al. 2015 | 1 | 1 | 1 | 1 | 1 | 1 | 1 | 1 | 8 |
| Ma, X. et al. 2007 | 1 | 1 | 1 | 1 | 1 | 1 | 1 | 1 | 8 |
| Ren, C. J. et al. 2016 | 1 | 1 | 0 | 0 | 1 | 0 | 1 | 0 | 4 |
| Lee, S. et al. 2009 | 1 | 1 | 0 | 0 | 1 | 1 | 1 | 1 | 6 |
| Fang, X. et al. 2011 | 1 | 1 | 1 | 1 | 1 | 1 | 1 | 1 | 8 |
| Liao, Z. H. et al. 2014 | 1 | 1 | 1 | 0 | 1 | 1 | 1 | 0 | 6 |
| Wang, W. Q. et al. 2013 | 1 | 1 | 1 | 1 | 1 | 1 | 1 | 1 | 8 |
| Zhao, Z. H. et al. 2009 | 1 | 1 | 1 | 0 | 1 | 1 | 1 | 1 | 7 |
| Ou, Q. M. et al. 2016 | 1 | 1 | 1 | 0 | 1 | 0 | 1 | 0 | 5 |
| Duan, W. D. et al. 2010 | 1 | 1 | 0 | 0 | 1 | 1 | 1 | 0 | 5 |
| Wei, B. et al. 2011 | 1 | 1 | 1 | 1 | 1 | 1 | 1 | 1 | 8 |
| Yue, L. L. et al. 2019 | 1 | 1 | 1 | 0 | 1 | 1 | 1 | 1 | 7 |
| Cui, L. J. et al. 2007 | 1 | 1 | 1 | 0 | 1 | 1 | 1 | 1 | 7 |
| Zhang, Y. S. et al. 2019 | 1 | 1 | 1 | 1 | 1 | 1 | 1 | 1 | 8 |
| Zhang, X. F. et al. 2015 | 1 | 1 | 0 | 0 | 1 | 1 | 1 | 0 | 5 |
| Peng, C. Q. et al. 2010 | 1 | 0 | 0 | 0 | 1 | 0 | 1 | 0 | 3 |
| Gui, L. H. et al. 2009 | 1 | 1 | 0 | 1 | 1 | 1 | 1 | 1 | 7 |
| Li, H. et al. 2009 | 1 | 1 | 1 | 0 | 1 | 0 | 1 | 0 | 5 |
| Hu, B. et al. 2003 | 1 | 1 | 1 | 1 | 1 | 1 | 1 | 0 | 7 |
| Wang, Z et al. 2017 | 1 | 1 | 1 | 0 | 1 | 1 | 1 | 1 | 7 |
| Zhang, S. J. et al. 2008 | 1 | 1 | 1 | 1 | 1 | 1 | 1 | 0 | 7 |
| Jacob, L. et al. 2019 | 1 | 1 | 1 | 0 | 1 | 1 | 1 | 0 | 6 |
| Cui, G. W. et al. 2019 | 1 | 1 | 0 | 0 | 1 | 1 | 1 | 1 | 6 |
| Li, Y. H. et al. 2018 | 1 | 1 | 1 | 1 | 1 | 1 | 1 | 0 | 7 |
| Zhao, N et al. 2018 | 1 | 1 | 1 | 1 | 1 | 1 | 1 | 1 | 8 |
| Ge, M. H. et al. 2018 | 1 | 1 | 1 | 0 | 1 | 1 | 1 | 1 | 7 |
| Dong, A. L. et al. 2008 | 1 | 1 | 1 | 0 | 1 | 0 | 1 | 0 | 5 |
| Xiao, L. et al. 2000 | 1 | 1 | 1 | 0 | 1 | 0 | 1 | 0 | 5 |
| Guo, K. F. et al. 2005 | 1 | 1 | 1 | 0 | 1 | 0 | 1 | 0 | 5 |
| Shi, J. J. et al. 2015 | 1 | 1 | 0 | 0 | 1 | 1 | 1 | 1 | 6 |
| Liu, D. M. et al. 2012 | 1 | 1 | 1 | 0 | 1 | 1 | 1 | 1 | 7 |
| Chen, Z. et al. 2017 | 1 | 1 | 0 | 1 | 1 | 1 | 1 | 0 | 6 |
| Liu, L. et al. 2011 | 1 | 1 | 1 | 1 | 1 | 1 | 1 | 1 | 8 |
| Yao, J. et al. 2010 | 1 | 1 | 1 | 0 | 0 | 1 | 1 | 1 | 6 |
| Xu, L. et al. 2018 | 1 | 1 | 1 | 0 | 1 | 1 | 1 | 1 | 7 |
| Zhao, Y. Z. et al. 1986 | 1 | 1 | 0 | 0 | 1 | 1 | 1 | 0 | 5 |

**Supplementary Table 2. Meta regression on logit prevalence of MDD in China (Method of moments).**

| Prevalence of MDD | Factors | Slope (*r*) | Intercept | Significance |
| --- | --- | --- | --- | --- |
| Lifetime prevalence | Survey year | 0.125 | －255.10 | *p* < 0.001 |
|  | Male percentage | －7.16 | －0.54 | *p* = 0.01 |
|  | Quality score | 0.23 | －5.47 | *p* = 0.009 |
|  | Mean age | －0.01 | －3.25 | *p* = 0.73 |
| Twelve-month prevalence | Survey year | 0.01 | －23.59 | *p* = 0.89 |
|  | Male percentage | －1.10 | －3.55 | *p* = 0.76 |
|  | Quality score | 0.07 | －4.47 | *p* = 0.80 |
|  | Mean age | －0.07 | －1.23 | *p* < 0.001 |
| Point prevalence | Survey year | 0.09 | －176.13 | *p* < 0.001 |
|  | Male percentage | －3.86 | －2.63 | *p* = 0.13 |
|  | Quality score | 0.11 | －5.23 | *p* = 0.25 |
|  | Mean age | 0.03 | －5.85 | *p* = 0.19 |

**Supplementary Figure 1. Funnel plot of the 27 included studies reporting lifetime prevalence of MDD in China. (Plotted by random effects)**

Egger’s test: *t* = 4.17, *p*＜0.001

**Supplementary Figure 2: Trim-and-fill method imputed lifetime prevalence of MDD in China.**

**Supplementary Figure 3. Funnel plot of the 11 included studies reporting 12-month prevalence of MDD in China. (Plotted by random effects)**

Egger’s test: *t* = 2.62, *p* = 0.031

**Supplementary Figure 4: Trim-and-fill method imputed 12-month prevalence of MDD in China.**

**Supplementary Figure 5. Funnel plot of the 29 included studies reporting point prevalence of MDD in China. (Plotted by random effects)**

Egger’s test: *t* = 3.69, *p* = 0.001

**Supplementary Figure 6: Trim-and-fill method imputed point prevalence of MDD in China.**
